# Factors associated with depression among women living with HIV and cervical cancer in Tanzania: a cross-sectional study

**DOI:** 10.3332/ecancer.2026.2128

**Published:** 2026-05-26

**Authors:** Alita Mrema, Queen Tarimo, Salum J Lidenge, Rehema Shungu, Herriethsiah Noah

**Affiliations:** 1 Department of Clinical Oncology, Muhimbili University of Health and Allied Sciences (MUHAS), PO Box 65001, Dar es Salaam, Tanzania; 2 Department of Clinical Oncology, Ocean Road Cancer Institute (ORCI), PO Box 3592, Dar es Salaam, Tanzania; 3 Department of Hematology, Muhimbili University of Health and Allied Sciences (MUHAS), PO Box 65001, Dar es Salaam, Tanzania

**Keywords:** cervical cancer, HIV, depression, social support, coping, Tanzania

## Abstract

**Background:**

Cervical cancer (CC) remains the leading cause of cancer-related mortality among women in sub-Saharan Africa, disproportionately affecting women living with HIV (WLWH). Depression is common in both HIV and cancer populations, but the psychological burden among women with dual diagnoses is poorly studied in Tanzania. This study investigated socio-demographic, clinical and psychological factors associated with depression in WLWH and CC.

**Methods:**

A cross-sectional study was conducted among 160 WLWH and CC, at least 3 months post CC treatment, between January and June 2024. Socio-demographic and clinical data were obtained from structured questionnaires and medical records. Depression prevalence and severity were assessed using the Patient Health Questionnaire. Perceived social support, HIV-related stigma and coping strategies were also measured using the tools were used in previous studies in Tanzania with good internal consistency. Multivariable linear regression was performed to identify independent predictors of depression.

**Results:**

The median age of participants was 49 years (IQR: 10). Most were married (91.9%), unemployed (51.9%) and had primary education (90%). Older age (51–73 years) was significantly associated with higher depression scores compared to younger participants (p < 0.001). Poor perceived social support was a strong predictor of depression (p = 0.0007). Negative self-image, as a component of HIV stigma, independently increased depression severity (p < 0.001). Conversely, active coping strategies were protective, with lower depression scores observed among women employing adaptive coping mechanisms (p = 0.018). Other socio-demographic and clinical characteristics, including education, marital status, CD4 count and cancer stage, were not significantly associated with depression.

**Conclusion:**

Depression is common among WLWH and CC in Tanzania, with older age, poor social support and negative self-image identified as significant risk factors, while active coping appeared protective. Integrating psychological screening, counseling and psychosocial interventions into routine oncologic and HIV care is critical to improve overall wellbeing and treatment outcomes in this vulnerable population.

## Introduction

Despite being highly preventable and treatable, cervical cancer (CC), remains the fourth most common cause of cancer-related deaths [1]. CC accounts for approximately 604,000 new cancer cases and 342,000 cancer deaths globally [1]. The incidence and mortality for CC in East Africa are high, with rates reported at 40.1/100,000 and 26.8/100,000, respectively [2]. The Africa region south of the Sahara, bears the highest burden of patients with CC, this being the leading diagnosis and cause of cancer-related mortality among women in Tanzania [2]. Women living with HIV (WLWH) are four to five times more likely to develop CC than the general population [3]. The co-infection of HIV with CC exacerbates disease progression and complicates HIV treatment outcomes [4]. Depression is a common mental illness that can substantially affect the quality of life of patients and treatment outcomes [5]. It is characterised by sadness, loss of interest or pleasure, feelings of guilt or low self-worth, disturbed sleep or appetite, tiredness and poor concentration [5]. Persons with chronic life-threatening illnesses like cancer are, in general, more likely to experience depression compared to non-cancer patients, with an estimated prevalence of 27% [6, 7]. Similarly, depression in people living with HIV (PLWH) is 2–3 times higher than in HIV-negative counterparts, and this has been linked to social stigma, loss of social support, decreased self-esteem and loneliness [6]. A study conducted in Ethiopia [8] reported a 32.5% depression prevalence among PLWH accessing care, while a similar study conducted in Tanzania, in newly diagnosed persons with HIV, prevalence of significant depression severity was reported at 41% [9]. These findings highlight the significant burden of depression among individuals living with HIV.

Higher depression severity scores among patients with CC have been linked to a number of socio-demographic characteristics, such as single motherhood, not living with a partner or divorce, financial difficulties, particularly in clients with low income, unemployment and lower educational status [8, 10]. Also, clinical characteristics of the patient, such as the stage of HIV, CD4 counts less than 200 cells/μL, presence of opportunistic infections and advanced International Federation of Gynecology and Obstetrics stage, were more likely to associate with depression [8, 10]. Regarding women's perceived social support, a significant negative correlation was reported between perceived poor social support and the risk of having depression [8].

Beyond these clinical and demographic determinants, the psychosocial dimensions of living with comorbid HIV and CC are particularly relevant. Women may face challenges related to body image changes following surgery and radiation therapy, fertility and reproductive identity concerns, sexual dysfunction and altered self-concept [11]. Such issues may influence treatment decision-making, psychological vulnerability, increase the risk of depression but also negatively impact the overall quality of life.

Despite compelling evidence that demonstrates elevated risk of depression in both PLWH and patients with cancer, there remains a critical knowledge gap regarding the compounding psychosocial burden that women face when confronted with concurrent HIV and CC, particularly in low-resource settings like Tanzania. This study, therefore, addressed this critical gap by evaluating the factors associated with depression among WLWH and Ass CC, including socio-demographic and clinical parameters, among women who have completed cancer treatment at Ocean Road Cancer Institute (ORCI) in Tanzania.

## Material and methods

### Study design

This was a cross-sectional study conducted at ORCI.

### Study setting

ORCI is a specialised government tertiary facility for cancer management in Dar es Salaam, Tanzania. ORCI receives all referred cases with cancer diagnoses from government and private hospitals in Tanzania and from neighbouring countries. The institute (ORCI) provides care to approximately 4,000 new cancer cases annually. CC is the leading malignancy managed at ORCI, with between 1,000 and 2,000 new cases per annum. Approximately 20% of newly diagnosed CC are also diagnosed in PLWH. ORCI offers cancer care services, including prevention, diagnostic, treatment, palliative and HIV care at HIV care and treatment clinic.

### Study population

The study involved WLWH and CC attending ORCI from January to June 2024. Women who were included in the study were those who were at least 3 months post cancer treatment, aged 18 years or older and provided informed consent to participate in the study. Less than 5% of patients who were approached for enrolment declined to participate in the study. Three patients were excluded due to poor performance status resulting in a total of 160 patients that qualified for final analysis.

### Participant recruitment

Study research assistants identified eligible 176 study participants from the Department of Medical Records. Patients who had both CC and HIV, and who were at least 3 months post-cancer treatment. Information about this study was provided to eligible clients, using their clinic-based contact mobile phone numbers and those expressing willingness to participate were invited to come to the hospital for more information about the study. 170 Study participants were willing to participate but 8 had financial constraints and 2 had poor performance status and could not make it to the study site. A total of 160 were enrolled for the possible consent process and an interview.

### Data collection

Trained nurses conducted face-to-face interviews with consented study participants. Socio-demographic information, clinicopathological and psychological health information from 160 consented women were abstracted from medical charts and structured questionnaires. Information on HIV viral load and CD4 counts were obtained from patients’ medical charts. (Most recent HIV viral load within the past 12 months and CD4 count in the past 6 months).

The Patient Health Questionnaire (PHQ-9) was used to measure depression severity. The adapted Swahili PHQ-9 has been validated in Tanzanian outpatients with an optimal cut-off score for probable depression of nine and above (representing moderate to severe Depression). It has a specificity of 87% and sensitivity of 78% with good internal consistency and Cronbach's alpha of 0.83 [12].

Perceived social support was measured using the ten-item Swahili version derived from UNC Functional Social Support Scale. Test-retest reliability for the UNC Functional Support Scale was reported to be 0.66 and the scale showed to have positive correlations with other social support measures [13]. It has been successfully used with good reliability with Cronbach's alpha value of 0.9 among PLWH in Tanzania. A mean score of <3 Across all ten items is scored as 'low social support' [14].

The Short Berger Scale was used to assess HIV Stigma. The 12-item scale consists of four domains with three questions in each sub-scale covering: disclosure concerns, personalised stigma, negative self-image and concerns with public attitudes. The scale has good reliability and Cronbach's α for all subscales was above 0.7 [15]. The questionnaire was translated into local language (Swahili) and administered face-to-face to patients. The total score on this scale ranges from 12 to 48, and a higher score indicates a more severe stigma [15].

The Brief COPE Questionnaire, developed by Carver *et al* [16], was utilised to assess participants' coping mechanisms. This questionnaire consists of 28 items that evaluate 14 different coping strategies. It is designed to assess both active and passive coping responses to challenging life events like Cancer and HIV. This tool has been used in Tanzania, showing acceptable internal consistency for active coping (Cronbach's *α* = 0.86) and avoidant coping (Cronbach's *α* = 0.62) [17]. The Brief COPE scales are categorised into two coping styles based on the framework by this tool was guided by the Lazarus and Folkman Transactional Model of Stress and Coping, which shows psychological stress arises from an individual’s evaluation of environmental challenges and their perceived capacity to manage those challenges. The model highlights the processes of primary appraisal, secondary appraisal, and coping responses, which may be either problem-focused or emotion-focused [18], problem-focused coping (including Religion, Acceptance, Planning, Positive Reframing, Active Coping, Instrumental Support, Emotional Support and Humor) and emotion-focused coping (comprising Self-Distraction, Denial, Venting, Behavioural Disengagement, Self-Blame and Substance Abuse). The total score is calculated by summing the scores of each subscale [16].

### Statistical analysis

Continuous variables were summarised as means and standard deviation, and median and interquartile ranges (IQR) presented. Categorical variables were summarised as proportions. Multiple linear regression models were used to identify associations between depression scores and socio-demographic characteristics, patients’ clinicopathological features and psychological factors. In multiple linear regression, a *p*-value of <0.05 was considered statistically significant. Results are presented as *β*-coefficients with a 95% confidence interval with *p*-values. SPSS Version 21 was used for data analysis.

### Ethical consideration

Approval to conduct the study was obtained from the ORCI Academics Research Publications and the Ethics Review Committee (10/VOL XXI/170). The study was described to study participants and written informed consent was obtained from each patient. All information obtained from participants was identified by unique identification numbers and confidentiality was maintained throughout the study.

## Results

### Study participants (Descriptive summary)

The study included 160 participants with a median age of 49 years (IQR: 10). Over half 90(56.3%) were below 51 years. Most participants were ever married 147 (91.9%) and had primary education 144 (90%). Over half 83(51.9%) were unemployed, and only 11(6.9%) participants reported having health insurance. The median time since HIV diagnosis was 144 months (IQR: 120), and the median duration on ART was 132 months (IQR: 108). Most participants (97.5%) had a viral load ≤1,000 copies/mL, indicating effective viral suppression, while 56.9% of participants had CD4 counts of less than 500 cells/μL. The median time since the last dose of cancer treatment was 12 months (IQR:28). Regarding CC, early-stage cancers (1A, 1B, 2A, 2B) were more common (74% of study participants), while 26% had advanced stages (3A, 3B, 4A, 4B) (Table 1).

### Bivariete analysis of predictors

The bivariable analysis identified predictors with *p* < 0.2 for inclusion in multivariate analyses.

Older participants (51–73 years) reported higher depression scores compared to younger participants (29–50 years) (8.09 ± 0.41 versus 1.26 ± 0.36; *p* < 0.001). Education level was marginally associated with depression (*p* = 0.113) and was considered for inclusion in multivariate analysis.

Concurrent chemoradiotherapy and radiation (*p* = 0.144) and cancer stage (*p* = 0.207) showed marginal associations with depression severity. Other clinical variables, including CD4 count and months on treatment, were not significantly associated.

Lower perceived social support was strongly associated with higher depression scores (5.71 ± 0.502 versus 2.61 ± 0.529; *p* < 0.001). Higher overall HIV stigma (*p* = 0.0381) and greater negative self-image (*p* < 0.001) were also significant predictors of depression severity, while avoidant coping strategies were associated with lower depression scores (*p* = 0.029) (Table 2).

### Multivariable analysis

Older age (51–73 years) had higher depression scores compared to younger participants (7.45 ± 0.74 versus 1.70 ± 0.68; *p* < 0.001).

Cancer stage, months on treatment and concurrent chemoradiotherapy were not independently associated with depression.

Poor social support (<3) predicted higher depression scores than adequate/good support (5.53 ± 0.709 versus 3.62 ± 0.715; *p* = 0.0007). Negative self-image was also a strong predictor (*p* < 0.001), while active coping strategies were protective (*p* = 0.018); Table 3.

## Discussion

Despite the dual burden of HIV infection and CC in Tanzania, mental health issues among WLWH and CC are not well documented. In this study, we investigated socio-demographic, clinicopathological and psychological factors associated with depression among WLWH and CC post CC treatment at ORCI in Tanzania. While several significant predictors of depression were identified in the bivariable analysis, older age, low perceived social support and negative self-image remained significant predictors of depression in multi-variable analysis.

The risk of depression has been reported to increase with age in several studies [19, 20]. A meta-analysis by Cai *et al* [21] reported a 35.1% prevalence of depression among older adults, further supporting the association between older age and increased risk of depression. Comorbidities are some of the possible reasons for increased depression risk as someone ages. In this study, high depression scores were found among older WLWH and cancer. It is possible that because of the limited life span, older women struggle more while managing two comorbid conditions [19]. As described by Bury’s biographical disruption model, chronic illness can interrupt a person’s usual life roles and interfere with future plans [22]. This is also supported by illness identity theory, which suggests that emotional outcomes depend on how individuals integrate illness into their sense of self [23]. Older women may particularly be vulnerable when illness becomes part of their identity, which will then interfere with their daily functioning, thereby increasing depression risk.

Additionally, the social support system tends to decrease with age, especially for those who have lost their spouses or family members. Furthermore, decreased productivity in older age may contribute to feelings of hopelessness and depression [20]. Also, older patients tend to experience the disease longer, have a higher risk of metastasis and have more disabilities, which all contribute to depression [24].

Beyond age, other socio-demographic and clinical characteristics, such as the level of education, the study found no significant association with depression among WLWH and CC. This aligns to a study conducted in Tanzania on depression in WLWH [9]. However, these findings contrast to other studies where they reported a significant association between education and depressive symptoms, for example, in a meta-analysis [21] examining factors influencing depression among CC patients shows that low education is linked to higher depression risk. The possible explanations for this discrepancy could be the small sample size in our study and also most of our participants were predominantly in primary level of education, making it difficult to detect differences between the groups. Similarly, marital status, employment, health insurance status, CD4 count and stage of the disease were all not associated with depression in this study. This contrasts with findings from Ethiopia [8], where marital status and CD4 count were associated with depression among women with HIV infection. Several factors may explain the discrepancy. Our cohort consisted with WLWH and CC, in whom cancer-related stressors may overshadow the influence of HIV clinical markers such as CD4. In addition, marital status in our cohort may have not accurately reflected the actual level of social and psychological support in these women.

Social support is known to be associated with the risk of depression [25]. The presence of close family and friends who are engaged in care during chronic illnesses reduces the psychological burden of the illness to the one being cared for [26]. In this study, majority of the WLWH and CC reported poor social support that was associated with high depression scores. Chronic illnesses like cancer and HIV are known to cause fatigue and anxiety as time goes on. Long-term care of cancer and HIV patients often leads to stressful emotional and economic relationships between the patient and the caregiver. Loss of time for economic activities and declining resources over time contribute to the strained relationships leading to the feelings of neglect, abandonment and depression among the patients. These findings emphasise the importance of social support from partners, families and friends to mitigate symptoms of depression [26].

In addition to social support, our findings also highlighted that active coping strategies had a positive impact on the reduction of depression, emphasising the impact of adaptive coping mechanisms and resilience among WLWH and cancer. Additionally, negative self-image emerged as a significant predictor of increased depression in these women, highlighting its harmful psychological effect. According to the illness identity framework [23], negative self-image reflects difficulty in reconciling bodily changes, sexual identity and femininity with the presence of chronic illness, leading to heightened emotional distress. This finding is consistent with a study conducted by Kim *et al* [19], which showed that women with body image concerns were nearly twice as likely to report symptoms of depression compared to those without such concerns.

Women in this study experience overlapping and interacting forms of stigma related to HIV infection, CC, reproductive health and increasing age. Intersectionality theory proposes that these stigmas interact and reinforce one another rather than acting independently, resulting in greater psychological burden [27]. Within this context, negative self-image may reflect the internalisation of multiple stigmatised identities, which in turn contributes to increased vulnerability to depression.

During this study, several limitations were noted. Because of the cross-sectional nature of the study, we could not make a causal inference. Similarly, self-reported measures of depression, stigma and coping strategies were used instead of an objective measure that is free of any bias. Social desirability bias may have influenced participants’ responses, particularly for reporting active coping strategies. The absence of a pre-treatment psychological baseline also restricted our ability to determine whether depressive symptoms preceded the cancer diagnosis or treatment.

## Conclusion

To the best of our knowledge, this is the first study to examine the socio-demographic factors and psychological factors associated with depression among WLWH and cancer in Tanzania.

Despite some noted limitations in this study, these findings emphasise that while other socio-demographic and clinical factors may not be strong predictors of depression, older age, poor social support, negative coping strategies and negative self-image play a critical role in the mental health status of WLWH and CC. A holistic approach that integrates psychological, social and medical issues is therefore essential in improving the outcomes of these patients. Such an approach could include screening for depression in the routine clinics, access to professional counselling services and programs enhancing body self-image and self-esteem.

## List of abbreviations

CC, Cervical cancer; CI, Confidence interval; HIV, Human Immunodeficiency Virus; IQR, Interquartile range; ORCI, Ocean Road Cancer Institute; PHQ-9, Patient Health Questionnaire-9; RT, Antiretroviral therapy; SPSS, Statistical Package for Social Sciences; TLD, Tenofovir/Lamivudine/Dolutegravir; TLE, Tenofovir/Lamivudine/Efavirenz; WLWH, Women living with HIV.

## Conflicts of interest

The authors declare no competing interests.

## Funding

This research received no external funding.

## Consent for publication

Not applicable.

## Author contributions

Conception and design: AM, HN

Data collection and assembly: AM, RS, HN

Data analysis and interpretation: AM, SL, QT

Manuscript writing: All authors

Final approval of manuscript: All authors.

## Availability of data materials

The data presented in this study are available on reasonable request from the corresponding author.

## Figures and Tables

**Table 1.** Demographics and clinical profile of study participants, November 2023 to April 2024.

Characteristic	N = 160
**AGE IN YEARS, MEDIAN (IQR)**	49 (10)
**AGE GROUP, *N* (%)**	
29**–50 YEARS**	90 (56.3)
51**–73 YEARS**	70 (43.8)
**MARITAL** s**TATUS, *N* (%)**	
** EVER** m**ARRIED**	147 (91.9)
** NEVER** m**ARRIED**	13 (8.1)
**EDUCATION** l**EVEL, *N* (%)**	
** PRIMARY** l**EVEL**	144 (90.0)
** SECONDARY** l**EVEL**	16 (10.0)
**CURRENT** e**MPLOYMENT, *N* (%)**	
** FORMALLY** e**MPLOYED**	77 (48.1)
** UNEMPLOYED**	83 (51.9)
**PRIVATE** h**EALTH** i**NSURANCE, *N* (%)**	
** YES**	11 (6.9)
** NO**	149 (93.1)
**TIME** s**INCE HIV** d**IAGNOSIS (MONTHS), MEDIAN (IQR)**	144 (120)
**DURATION** o**N ART (MONTHS), MEDIAN (IQR)**	132 (108)
**CD4** c**OUNT, *N* (%)**	
≤**500**	91 (56.9)
**>500**	67 (41.9)
**MISSING**	2 (1.3)
**HIV** v**IRAL** l**OAD** w**ITHIN** p**AST 6** m**ONTHS (COPIES/M**L**), *N* (%)**	
≤**1**,000	156 (97.5)
>**1**,000	3 (1.8)
Missing	1 (0.6)
**ART** t**YPE, *N* (%)**	
**1ST** l**INE TLD**	157 (98.1)
T**ENOFOVIR/LAMIVUDINE/EFAVIRENZ (TLE)**	3 (1.9)
**MONTHS** s**INCE** l**AST** d**OSE OF** c**ANCER** t**REATMENT (MONTHS), MEDIAN (IQR)**	12 (28)
**CANCER** s**TAGE, *N* (%)**	
**1A, 1B, 2A, 2B**	119 (74%)
**3A, 3B, 4A, 4B**	41 (26%)

**Table 2.** Bivariable regression analysis of predictors of depression among study participants, November 2023 to April 2024 (N = 160).

	Least square mean QoL (SE)	p-value	95% CI
Age (years)		**< 0.001**	
29–50	1.26 (0.36)		(0.65–1.87)
51–73	8.09 (0.41)		(7.28–8.90)
CD4 count (cells/mm^3^)		0.7981	
≤500	4.15 (0.504)		(3.15–5.15)
>500	4.35 (0.596)		(3.17–5.53)
Employment status		0.4295	
Formally employed	4.55 (0.547)		(3.47–5.63)
Unemployed	3.94 (0.54)		(2.87–5.01)
Private health insurance		0.928	
Yes	4.23 (0.399)		(3.44–5.02)
No	4.36 (1.45)		(1.47–7.25)
Concurrent chemotherapy and radiation		**0.1443**	
Yes	5.7 (1.07)		**(3.59–7.81)**
No	4.02 (0.409)		**(3.21–4.83)**
Marital status		**0.2386**	
Never married	4.37 (0.399)		**(3.59–5.15)**
Ever married	2.67 (1.380)		**(0.02–5.32)**
Education		**0.1131**	
Secondary level education	4.44 (0.403)		**(3.65–5.23)**
Primary level education	2.44 (1.19)		**(0.07–4.81)**
Short berger HIV overall stigma score (per one point increase in stigma score)	4.24 (0.38)	**0.0381**	**(3.51–4.97)**
Disclosure concern	4.24 (0.384)	0.3085	(3.50–4.98)
Personalised stigma	4.24 (0.382)	**0.1167**	(3.51–4.97)
Public attitude concern	4.24 (0.385)	0.924	(3.51–4.97)
Negative self-image	4.24 (0.364)	**< 0.001**	(3.54–4.94)
UNC functional social support scale		**< 0.001**	
Poor social support (< 3)	5.71 (0.502)		**(4.72–6.7)**
Adequate/Good social support (≥3)	2.61 (0.529)		(1.56–3.66)
Brief COPE questionnaire score			
Avoidant coping (per one point increase in avoidant coping score)	4.24 (0.379)	**0.029**	**(3.51–4.97)**
Active coping (per one point increase in active coping score)	4.24 (0.382)	**0.1147**	**(3.51–4.97)**
Months on treatment (per month increase of treatment length)	4.24 (0.385)	0.8523	(3.51–4.97)
Cancer stage		**0.2074**	
1A, 1B, 2A, 2B	3.96 (0.442)		(3.08–4.84)
3A, 3B, 4A, 4B	5.08 (0.766)		(3.58–6.58)

*p* < 0.2 considederd for multivariable regression

**Table 3.** Final multivariable regression results for depression and associated predictors among study participants, November 2023 to April 2024 (N = 160).

	Least square mean QoL (SE)	p-value	95% CI
Age (years)		< 0.001*	
29–50	1.70 (0.68)		(0.37–3.03)
51–73	7.45 (0.74)		(6.00–8.9)
UNC functional social support scale		0.0007*	
Poor social support (<3)	5.53 (0.709)		(4.14–6.92)
Adequate/Good social support (≥3)	3.62 (0.715)		(2.22–5.02)
Months on treatment (per month increase of treatment length)	−0.03 (0.020)	0.119	(−0.07–0.01)
Cancer stage		0.141	
1B, 2A, 2B	4.11 (0.669)		(2.80–5.42)
3A, 3B, 4A, 4B	5.04 (0.783)		(3.51–6.57)
Brief COPE questionnaire score			
Avoidant coping (per one point increase in avoidant coping score)	4.57 (0.656)	0.192	(3.28–5.86)
Active coping (per one point increase in active coping score)	4.57 (0.656)	0.018	(3.28–5.86)
Concurrent chemotherapy and radiation		0.16	
No	5.12 (0.88)		(3.39–6.85)
Yes	4.03 (0.605)		(2.84–5.22)
Marital status		0.4326	
Never married	4.2 (1.02)		(2.20–6.20)
Ever married	4.95 (0.521)		(3.93–5.97)
Education		0.373	
Secondary level education	4.20 (0.932)		(2.37–6.03)
Primary level education	4.95 (0.593)		(3.79–6.11)
Personalised stigma	4.57 (0.656)		(3.28–5.86)
Negative self-image	4.57 (0.656)	< 0.001*	(3.28–5.86)
*p < 0.05 considered statistically significant			

## References

[1] Stelzle D, Tanaka LF, Lee KK (2021). Estimates of the global burden of cervical cancer associated with HIV. Lancet Glob Health.

[2] Bray F, Laversanne M, Sung H (2024). Global cancer statistics 2022: gLOBOCAN estimates of incidence and mortality worldwide for 36 cancers in 185 countries. CA Cancer J Clin.

[3] Mrema D, Ngocho JS, Mremi A (2023). Cervical cancer in Northern Tanzania—What do women living with HIV know. Front Oncol.

[4] Clifford GM, De Vuyst H, Tenet V (2016). Effect of HIV infection on human papillomavirus types causing invasive cervical cancer in Africa. J Acquir Immune Defic Syndr (1988).

[5] WHO (2017). Depression and Other Common Mental Disorders Global Health Estimates.

[6] Du X, Zhang Q, Hao J (2023). Global trends in depression among patients living with HIV: a bibliometric analysis. Front Psychol.

[7] Mejareh ZN, Abdollahi B, Hoseinipalangi Z (2021). Global, regional, and national prevalence of depression among cancer patients: a systematic review and meta-analysis. Indian J Psychiatry.

[8] Yousuf A, Musa R, Isa MLM (2020). Anxiety and depression among women living with HIV: prevalence and correlations. Clin Pract & EpidemiolMental Health.

[9] Madundo K, Knettel BA, Knippler E (2023). Prevalence, severity, and associated factors of depression in newly diagnosed people living with HIV in Kilimanjaro, Tanzania: a cross-sectional study. BMC Psychiatry.

[10] Golubovic ST, Binic I, Krtinic D (2022). Risk Factors and Predictive Value of Depression and Anxiety in Cervical Cancer Patients. Medicina (Lithuania).

[11] La Rosa VL, Shah M, Kahramanoglu I (2020). Quality of life and fertility preservation counseling for women with gynecological cancer: an integrated psychological and clinical perspective. J Psychosomatic Obstetrics Gynecology.

[12] Smith Fawzi MC, Ngakongwa F, Liu Y (2019). Validating the Patient Health Questionnaire-9 (PHQ-9) for screening of depression in Tanzania. Neurol Psychiatry Brain Res.

[13] Kaaya S, Siril H, Mcadam K (2020). Agents of change: comparing HIV-related risk behavior of people attending ART clinics in Dar es Salaam with members of their social networks. PLoS One.

[14] Antelman G, Smith Fawzi MC, Kaaya S (2001). Predictors of HIV-1 serostatus disclosure: a prospective study among HIV-infected pregnant women in Dar es Salaam, Tanzania. AIDS.

[15] Reinius M, Wettergren L, Wiklander M (2017). Development of a 12-item short version of the HIV stigma scale. Health Qual Life Outcomes.

[16] Carver CS (2006). Stress, coping, and health. Foundations of Health Psychology.

[17] Steglitz J, Ng R, Mosha JS (2012). Divinity and distress: the impact of religion and spirituality on the mental health of HIV-positive adults in Tanzania. AIDS Behav.

[18] Folkman S (2013). Stress: appraisal and coping. Encyclopedia of Behavioral Medicine.

[19] Kim SH, Kang S, Kim YM (2010). Prevalence and predictors of anxiety and depression among cervical cancer survivors in Korea. Int J Gynecological Cancer.

[20] Dhakal M, Basel P (2023). Prevalence of depression and associated factors among cervical cancer patients attending tertiary center in Bhaktapur, Nepal [Internet].

[21] Cai H, Jin Y, Liu R (2023). Global prevalence of depression in older adults: a systematic review and meta-analysis of epidemiological surveys. Asian J Psychiatry.

[22] Bury M (1982). Chronic illness as biographical disruption. Sociol Health Illn.

[23] Oris L, Luyckx K, Rassart J (2018). Illness Identity in Adults with a Chronic Illness. J Clin Psychol Med Settings.

[24] Ayalew M, Deribe B, Duko B (2022). Prevalence of depression and anxiety symptoms and their determinant factors among patients with cancer in southern Ethiopia: a cross-sectional study. BMJ Open.

[25] Alkubati SA, Halboup AM, Zoromba MA (2025). Prevalence and determinants of depression and its association with social support among cancer patients: implications for enhancing oncology care. BMC Psychol.

[26] Karawekpanyawong N, Kaewkitikul K, Maneeton B (2021). The prevalence of depressive disorder and its association in Thai cervical cancer patients. PLoS One.

[27] Turan JM, Elafros MA, Logie CH (2019). Challenges and opportunities in examining and addressing intersectional stigma and health BMC Med.

